# Light Photo Treatment at 405 nm Can Effectively Kill *Leishmania* Parasites

**DOI:** 10.3390/microorganisms14051135

**Published:** 2026-05-16

**Authors:** Ikeoluwa Adekoya, Michelle Maclean, Logan Mackie, Katharine C. Carter

**Affiliations:** 1 Strathclyde Institute of Pharmacy & Biomedical Sciences (SIPBS), University of Strathclyde, Glasgow G4 0RE, UK; ikehamzat@yahoo.com (I.A.); logan.mackie@strath.ac.uk (L.M.); 2 The Robertson Trust Laboratory for Electronic Sterilisation Technologies, Department of Electronic and Electrical Engineering, University of Strathclyde, Glasgow G1 1XW, UK; michelle.maclean@strath.ac.uk; 3 Department of Biomedical Engineering, University of Strathclyde, Glasgow G4 0NW, UK

**Keywords:** *Leishmania*, 405 nm light treatment, phototherapy, blue light therapy, antimicrobial light, cutaneous leishmaniasis

## Abstract

Cutaneous leishmaniasis is a protozoan disease which is responsible for significant morbidity in humans. Currently, there is no clinically approved vaccine to prevent infections, and, therefore, treatments to cure skin lesions are required. Ideally, a treatment that can be self-administered to affected areas is desirable. In this study, the effect of violet-blue light, of wavelength in the region of 405 nm, on the survival of *Leishmania major* and *Leishmania mexicana* was determined using *in vitro* and *in vivo* models. Light treatment caused significant killing of both promastigotes and intracellular amastigotes (*p* < 0.001) of both species *in vitro*, and *L. mexicana* intracellular amastigotes were more resistant to light treatment than *L. major* intracellular amastigotes. Treatment with violet-blue light at a dose of 45 J/cm^2^ (0.15 W/cm^2^ for 5 min) per day on days 3–7 post-infection in an *in vivo* footpad model caused a significant reduction in *L. major* parasite burdens on day 5 post-infection (*p* < 0.05) in one of two experiments, though by day 10 post-infection, parasite numbers had recovered to those of controls. The results of this study clearly demonstrate that violet-blue light can kill both *L. major* and *L. mexicana* parasites, but application to infected cutaneous tissues requires refinement.

## 1. Introduction

Cutaneous leishmaniasis (CL) is caused by infection with the protozoan parasite *Leishmania* and is transmitted by the female sand fly. It is endemic in several countries, and there are 0.6–1 million new cases/year [1]. The clinical symptoms of leishmaniasis depend on the infecting species, its virulence, host genetics, and the host’s immune response to the parasite [2,3]. Patients present with a single lesion at the insect bite site or multiple lesions when infected with Old World species (e.g., *L. tropica*, *L major*, *L. aethiopica*) or New World species (e.g., *L. mexicana*, *L. amazonesis*, *L. venezuelensis*, and *L. viannia* subgenera, including *L. V. braziliensis*, *panamensis*, *guyanesis*) [4]. The lesions can self-heal with time but may leave disfiguring scars on the individual, which can cause psychological harm and social exclusion [5]. It should be feasible to vaccinate against CL, but at present, there is no recommended vaccine candidate, although some are in clinical development, e.g., Leish-111f + MPL-SE vaccine [6] and ChAd63-KH [7]. Therefore, control of CL relies on treatment using chemotherapy or other methods such as cryotherapy, thermotherapy, or irradiation with ultraviolet (UV) light [8]. There are a limited number of drugs to treat CL, and most are given parenterally rather than by the topical route. Furthermore, an extended treatment regimen is required, often not only resulting in adverse clinical side effects but also the selection of drug-resistant parasites [9]. Another potential way to treat CL lesions locally is through use of non-UV-based optical therapy. Previous work has demonstrated the broad-spectrum antimicrobial efficacy of violet-blue light in the region of 405 nm [10], with its improved compatibility with mammalian cells and tissue due to these wavelengths being in the visible light region [11]. Microbial inactivation occurs via a photodynamic process which induces the production of reactive oxygen species (ROS), resulting in oxidative damage to the exposed microorganisms [12,13,14]. While numerous publications have highlighted the broad-spectrum efficacy of violet-blue light against a range of bacteria, endospores, yeast, fungi, and viruses, it is only recently that anti-parasite activity has been observed. Two recent studies have indicated that violet-blue light could inactivate *Leishmania donovani* promastigotes seeded in human blood plasma [15] and that blue light-emitting diode (LED) phototherapy was toxic to *Leishmania amazonensis*, *L. braziliensis*, and *L. infantum* [16]. In this study, we determined the feasibility of exposure to 405 nm violet-blue light as a therapeutic tool by measuring the survival of *L. major* and *L. mexicana* using both *in vitro* and *in vivo* models. Our studies demonstrated violet-blue light to be toxic to *Leishmania* parasites *in vitro* and highlighted factors that need to be considered for future *in vivo* use, including the infecting species.

## 2. Materials and Methods

### 2.1. Materials

L-NIL (N6-(1-iminoethyl)-L-lysine hydrochloride), an inhibitor of nitric oxide production, was obtained from Alfa Aesar (Lancashire, UK). Medium and supplements used for parasite culture, RPMI 1640, Foetal Calf Serum (FCS), Penicillin–Streptomycin, L-Glutamine, were obtained from Gibco BRP (Paisley, UK). D-Luciferin used for bioluminescence assay was supplied by Calliper Life Sciences (Runcorn, UK). Alamar blue reagent was purchased from Bio-Rad, Hercules, CA, USA. Antibiotics were supplied by Sigma Aldrich, Irvine, UK. All other reagents were of analytical grade.

### 2.2. Animals and Parasites and Ethical Approval

Female age-matched BALB/c (20–25 g) in-house inbred female mice from the University of Strathclyde colony, *L. mexicana* (strain *Lmex*luc, derived from MNYC/BZ/M379), and *L. major* (*Lmaj*luc, derived from WHOM/IR/173) were used in studies. All studies had local ethical approval and United Kingdom Home Office approval (project license PPL PP7245718).

### 2.3. L. major and L. mexicana In Vitro Promastigote Studies

**Figure 1:**
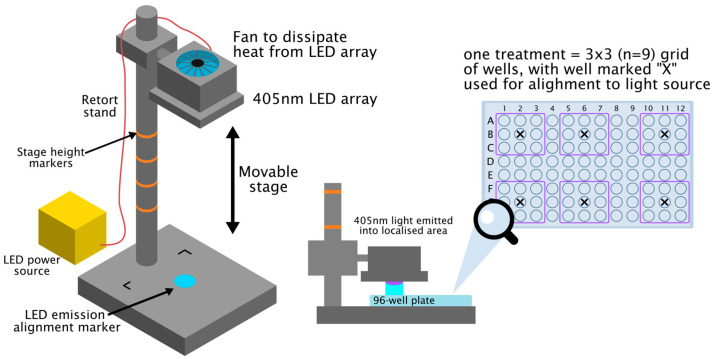
Experimental set up for 405 nm light exposure. A 405 nm light-emitting diode (LED) array was held on a retort stand with a movable arm. Samples were positioned directly below the LED array (using a marker on the stand base to ensure consistent alignment) and exposed to fixed irradiances of light (measured using a radiant power meter and photodiode detector). Multiple samples (*n* = 9) were individually added to a 96-well tissue culture treatment plate so that one plate could be used for up to 6 treatments in promastigote studies. A maximum of two treatments were added to a tissue culture plate for *in vitro* macrophage studies.

*L. major* or *L. mexicana* promastigote cultures were set up from parasites stored in freezing medium (8% *v*/*v* DMSO in FCS) at −80 °C. The cryoculture (approx. 1 mL, approximately 1 × 10^8^ parasites) was gently defrosted and added to 10 mL of incomplete medium (RPMI 1640 supplemented with 100 μg/mL penicillin/streptomycin and 2 mM L-glutamine) with mixing. The parasites were pelleted by centrifuging at 300 *g* for 5 min. The parasite pellet was resuspended in 20 mL of complete medium (incomplete medium supplemented with 10% FCS *v*/*v*), transferred to a 30 mL tissue culture flask, and incubated at 26 °C until the parasites reached stationary phase. The parasites were then pelleted, resuspended in fresh complete medium, and passaged for future studies or resuspended in freezing medium and stored at −80 °C. Aliquots of a promastigote suspension (100 µL, in complete medium or saline, 5 × 10^5^/well, *n* = 9) were added to the appropriate wells of a 96-well cell culture plate under aseptic conditions, and the plate was positioned under a 405 nm light source. The light source used was a 405 nm light-emitting diode (LED) array (ENFIS PhotonStar Innovate UNO 24, PhotonStar Technologies Ltd., Romsey, UK) powered by a 62 V LED driver (Philips, Eindhoven, The Netherlands), with the array mounted using a polyvinyl chloride (PVC) housing on a retort stand which held the array above a PVC base plate, with a marker on the base plate used to align the samples directly below the source (as detailed in Figure 1). The irradiance (W/cm^2^) levels used for sample exposure were determined using a radiant power meter and photodiode detector (LOT-Oriel Ltd., Stratford, CT, USA), and the treatment dose was calculated using the following equation: Dose (J/cm^2^) = irradiance (W/cm^2^) × time (s), with the irradiance being a mean reading from 9 adjacent wells (Figure 1). Readings from the radiant power meter and photodiode detector indicated that 9 adjacent wells in a 96-well plate received the same light dose using the system shown in Figure 1. Exposure to an irradiance of 0.11 W/cm^2^ for 5, 10, or 15 min would give a light dose equivalent to 33, 66, or 99 J/cm^2^ respectively. Treated cells were then either transferred directly to the appropriate wells of a 96-well cell culture plate (100 µL, *n* = 9, complete medium parasite suspension) or centrifuged (300 *g*) and resuspended in 0.9 mL of fresh complete medium and then added to the appropriate wells of a 96-well culture plate (100 µL, *n* = 9). Control samples were prepared in separate plates to ensure no exposure to 405 nm light. Plates were then incubated at 26 °C to allow the parasites to grow. At 24 h post-treatment, 20 µL of luciferin stock solution in PBS pH 7.4 was added to each sample (final luciferin concentration 150 µg/mL), and the bioluminescent signal (medium binning) emitted by each sample was determined using the 8 × 12 grid provided by the system software and automatic setting. The effect of light treatment on parasite survival was determined by calculating the suppression in the bioluminescent signal of treated samples compared to the mean control value. Nine replicates were used for each treatment and control condition, and experiments were repeated at least three times to show that results were consistent. Figure S1 (Supplementary Data) shows the type of images obtained *in vitro* parasite studies.

### 2.4. L. major and L. mexicana In Vitro Amastigote Studies

Bone-marrow-derived macrophages were obtained using the method detailed in previous studies [17]. Bone-marrow-derived macrophages (100 µL, 0.5 × 10^5^ in complete medium) were added to the appropriate wells of a 96-well tissue culture plate (either one treatment/plate or a treated group and control group on the same plate) and incubated for 3 or 24 h at 37 °C in an atmosphere of 95% air/5% CO_2_. The macrophages were then infected with *L. major* or *L. mexicana* using a 10:1 parasite:host cells ratio and incubated for a further 24 h. The medium was then removed from the cells and replaced with 100 µL of saline or complete medium so that any free parasites were removed. The infected macrophages (*n* = 9) were then treated with blue light for 5, 10, or 15 min using an irradiance of 0.11 W/cm^2^. Thus, cells were treated with a light dose equivalent to 33, 66, or 99 J/cm^2^, respectively, using the system shown in Figure 1. The saline or medium was then removed and replaced with 100 µL of complete medium and the plate was then incubated as before for a further 48 h. Controls were similarly treated without exposure to light treatment. Luciferin stock solution (20 µL in PBS pH 7.4, final concentration 150 µg/mL) was added to each sample at the end of the incubation period, and the bioluminescent signal (medium binning) emitted by each sample was determined using the 8 × 12 grid provided by the system software and automatic setting. The effect of treatment on parasite growth was determined by calculating the mean suppression in the bioluminescent signal of treated samples (*n* = 9) compared with the mean control value. All experiments were repeated at least 3 times to ensure that results were consistent.

The effect of treatment on uninfected bone-marrow-derived macrophage viability was determined using an AlamarBlue assay to assess whether killing of *Leishmania* amastigotes involved host cell cytotoxicity [18]. Uninfected macrophages were plated out as above (Figure 1, 100 µL, 0.5 × 10^5^ in complete medium, two groups/plate, *n* = 9/treatment) and the plate was incubated overnight at 37 °C in an atmosphere of 95% air/5% CO_2_. The medium was removed from each sample and replaced with 100 µL of saline. Treated cells (*n* = 9) were exposed to 405 nm light for 15, 10, or 5 min, equating to 99, 66, and 33 J/cm^2^ irradiance light doses. The saline was then removed from samples and replaced with 100 µL of complete medium. Control and treated cells were incubated as before for a further 40 h, and then 10% *v*/*v* AlamarBlue reagent was added to each sample. The absorbance of the samples was measured at 48 h post-exposure at 570 nm and 600 nm using a Spectramax 190° spectrophotometer (Molecular Devices, San Jose, CA, USA). The percentage reduction of alamarBlue from the blue resazurin compound to a red resorufin compound was determined according to the manufacturer’s instructions (https://www.bio-rad-antibodies.com/measuring-cytotoxicity-proliferation-spectrophotometry-fluorescence-alamarblue.html, accessed on 25 January 2026). The mean control data was used to determine the percentage reduction in viability for treated samples (*n* = 9) compared to the mean control value. All experiments were repeated at least 3 times to ensure that results were consistent.

### 2.5. L. major In Vivo Studies

A sample size of 5 mice/group was used in *in vivo* studies, as data from previous studies have shown that 5 animals/group is required to detect a significant difference at *p* < 0.05 using an 80% power. BALB/c female mice (*n* = 5/treatment) were infected by subcutaneous injection into the footpad without anesthesia with incomplete medium containing 1 × 10^7^/mL *L. major* luciferase-expressing promastigotes (*Lmaj*Luc). Mice were given light treatment once/day for 5 consecutive days, using the custom-built apparatus shown in Figure 1, starting on day 3 post-infection. Consequently, each mouse was treated individually so they could be closely monitored for any adverse effects during treatment. Pilot studies were carried out using four *L. major*-infected mice (two control and two treated mice). The effect of light treatment on the behavioral response of mice, i.e., involuntary movement of the foot due to discomfort, and skin temperature readings were used to select the light dose given. The hypothesis being tested was that light treatment would cause a significant reduction in parasite burdens, and this would be demonstrated as a significant reduction in the bioluminescent signal in treated animals compared with controls and/or a significant reduction in footpad size compared with controls. Mice were treated once/day for 5 consecutive days, as this seemed a reasonable time for the light treatment to have an effect and meant we did not exceed the limit of anesthesia exposure for our U.K. Home Office animal project license. Mice were treated once/day for 5 min on days 3–8 post-infection with a dose of 0.15 W/cm^2^ to give a dose of 45 J/cm^2^/day. Mice were anesthetized using inhaled anesthesia, and areas of the body not to be treated (e.g., tail) were covered with drape material to prevent light exposure.

The effect of treatment on parasite growth was monitored by measuring the change in footpad thickness of the uninfected and infected footpad of each control and treated mouse using a pocket thickness gauge range of 9 mm (Mitutoyo Corporation, Tokyo, Japan) at the start and end of the study. In addition, parasite growth was also monitored over the course of the study by measuring the difference in bioluminescence emitted by the infected and uninfected footpad of each control and treated mouse over the course of the study [19]. The same-sized region of interest was used for the footpad of each mouse at each time point. The amount of bioluminescence (BLI) emitted in each region of interest (ROI) was determined using the Living Image software Version 4.8.4, and the results were recorded as photons/sec emitted. The parasite-specific bioluminescent signal for each infected mouse was determined by subtracting the bioluminescent signal of the uninfected footpad from the signal emitted by the infected footpad for each mouse. Light treatment is known to be associated with the generation of heat; therefore, the temperature of the treated footpad was determined immediately before and after treatment using a thermocouple (Thermometer DT-610B, ATP, K type, RS stock 180–540 thermocouple). The effect of heating the infected footpad to 37 °C on parasite survival was determined by placing the infected footpad of an anesthetized mouse (inhaled anesthesia) in a water bath at 37 °C for 5 min/day on days 3–7 post-infection to mimic the effects of the heat generated by light treatment in some studies. The effect of treatment on parasite growth was also determined as above for each mouse. Experiments were repeated more than once to show that significant results were consistent. Figure S2 (Supplementary Data) shows the type of images obtained in *in vivo* parasite studies.

### 2.6. Statistical Analysis of Data

Data from *in vitro* and *in vivo* experiments were analyzed using a Mann–Whitney U test for two groups or a Kruskal–Wallis test followed by Dunns post hoc test when more than two groups were used in studies. Results were considered statistically significant at a *p* value < 0.05.

## 3. Results

### 3.1. Photo-Treatment Had a Dose-Dependent Effect on the Survival of Leishmania Promastigotes

**Figure 2:**
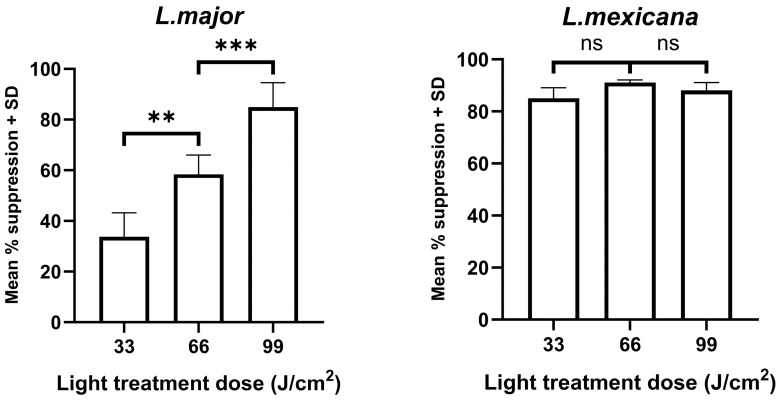
The effect of 405 nm photo-treatment on the survival of *L. major* and *L. mexicana* promastigotes. Promastigotes (*n* = 9) were exposed to light with average irradiance of 0.11 W/cm^2^ for 5, 10 or 15 min to give a light dose equivalent to 33, 66, or 99 J/cm^2^, respectively. Light-treated and non-treated (control) parasites were then incubated at 26 °C for 24 h and the amount of bioluminescence emitted/sample was determined. The mean suppression in bioluminescence for treated cells compared with the mean control group was also determined. *** *p* < 0.001, ** *p* < 0.01 for relevant groups, ns—not significant.

Treatment with a light dose of 33 J/cm^2^ caused a significant reduction in *L. major* parasite growth, based on the bioluminescence emitted by samples, compared with control values (33.7% suppression; *p* < 0.01). Increasing the light dose given significantly enhanced parasite killing, with the highest dose causing the greatest suppression in parasite numbers compared with controls (*p* < 0.001, Figure 2).

*L. mexicana* promastigotes were more sensitive to light treatment, as all three doses caused significant parasite killing, with the lowest dose of 33 J/cm^3^ causing a significant reduction in parasite burdens compared to control values (*p* < 0.01, Figure 2). However, a dose-dependent effect did occur in a subsequent experiment (mean % suppression compared to control values, 33 J/cm^2^ 44 ± 5, 66 J/cm^2^ 73 ± 16, 99 J/cm^2^ 90 ± 2).

**Figure 3:**
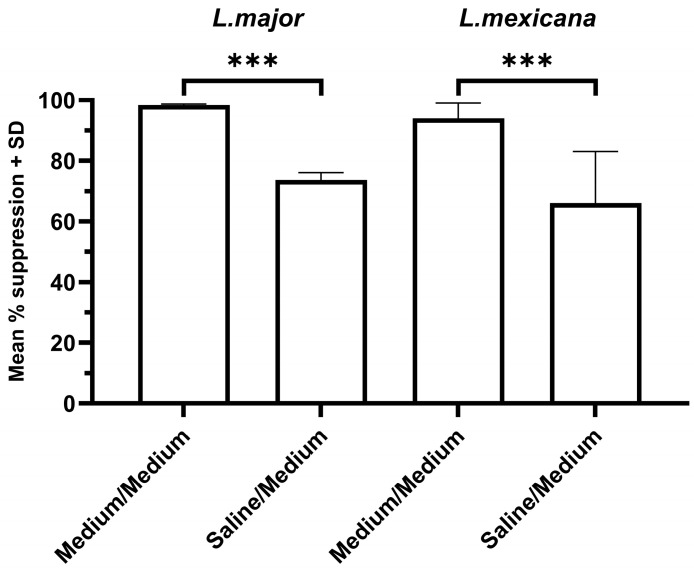
The effect of 405 nm photo-treatment on the survival of *L. major* and *L. mexicana* promastigotes. Promastigotes (*n* = 9) were suspended in saline or medium and then treated with a light dose of 66 J/cm^2^ (0.11 W/cm^2^, 10 min). The parasites were then resuspended in complete medium and incubated at 26 °C for 24 h and the amount of bioluminescence emitted/sample for treated and untreated control samples was determined. The mean suppression in bioluminescence was then calculated for treated samples compared to the mean value of the untreated control, *** *p* < 0.001.

As the complete media used in this study contain a variety of photo-sensitive constituents, these may interact with the light photons and contribute to the generation of reactive oxygen species (ROS) during photo-treatment. Therefore, a comparison of the antimicrobial efficacy when suspended in saline (which contains no photosensitive components) was conducted to identify any potential enhancement caused by exposure in the complete medium. Preliminary studies showed that parasites did not grow well if maintained in saline for 24 h after treatment; therefore, parasites exposed in saline were resuspended in complete medium after light treatment (termed ‘saline/medium’ treatment). The results showed that for both *L. major* and *L. mexicana*, successful parasite suppression was achieved when parasites were suspended in saline rather than medium; however, this suppression was significantly less than that achieved when promastigotes were exposed in complete medium (*p* < 0.001, Figure 3). These results indicate that significantly enhanced inactivation of the parasites can be achieved when exposed in culture medium, likely due to the cytotoxicity of additional ROS produced from the photoexcitation of the suspending media.

### 3.2. Photo-Treatment Had a Dose-Dependent Effect on the Survival of Intracellular Leishmania Amastigotes

**Figure 4:**
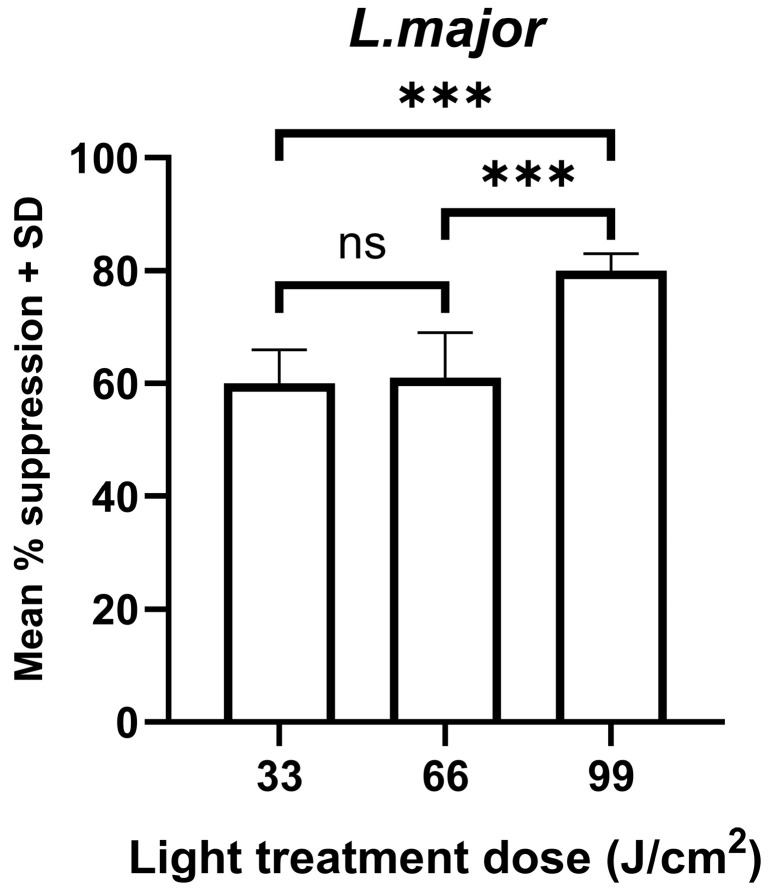

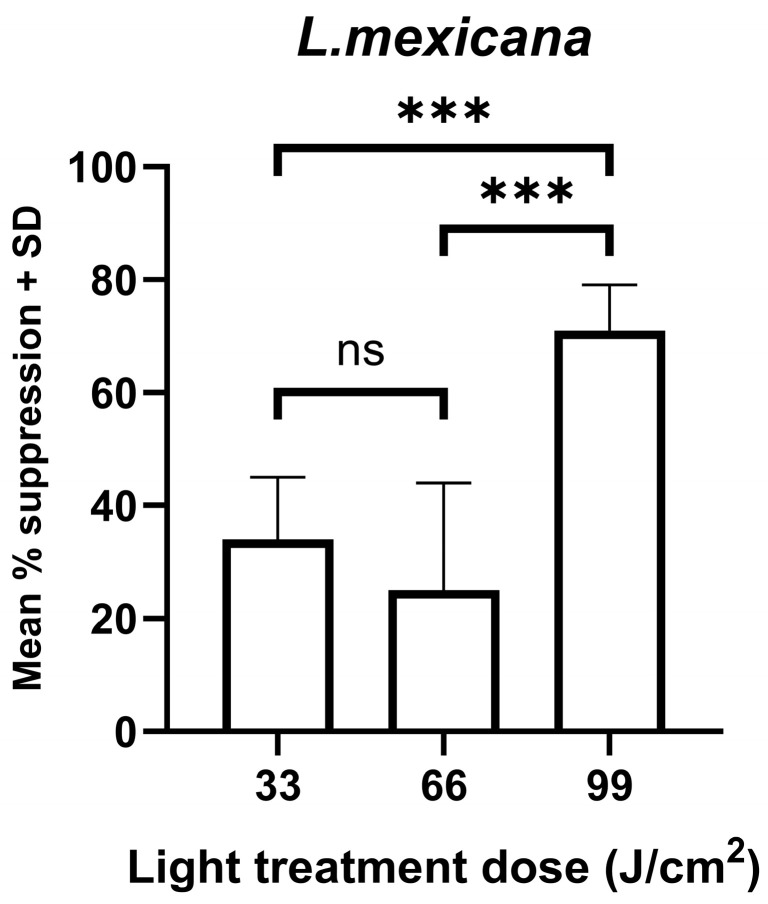
The effect of varying doses of 405 nm photo-treatment on the survival of *L. major* amastigotes. *L. major*- or *L. mexicana*-infected macrophages (*n* = 9) were exposed to 0.11 W/cm^2^ light treatment for 5, 10, and 15 min (equivalent to doses of 33, 66, and 99 J/cm^2^, respectively) and then cultured along with controls at 37 °C for 48 h. A treated and control group were present on each plate. The amount of bioluminescence emitted per sample was determined and used to calculate the mean suppression in bioluminescence compared to the mean control value (*n* = 27). *** *p* < 0.001 for relevant groups, ns—not significant.

*Leishmania* promastigotes transform to the intracellular amastigote stage within the mammalian host. Therefore, it is important to demonstrate that this stage of the life cycle is also susceptible to light treatment. The results showed that 405 nm light treatment had a dose-dependent effect on the survival of intramacrophage amastigotes for both *L. major* and *L. mexicana*, based on the amount of bioluminescence emitted by the samples (Figure 4).

**Figure 5:**
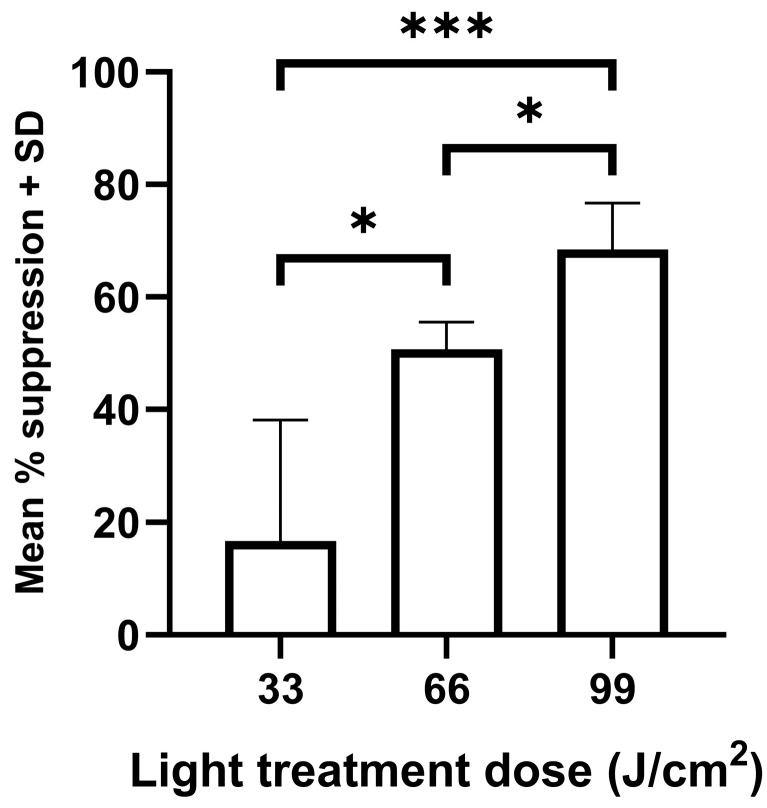
The effect of varying doses of 405 nm photo-treatment on the viability of uninfected macrophages. Cells (*n* = 9) were exposed to 0.11 W/cm^2^ light treatment for 5, 10, and 15 min (equivalent to doses of 33, 66, and 99 J/cm^2^, respectively) and then cultured along with controls at 37 °C for 48 h. An Alamar blue viability assay was used to compare the viability of treated and control cells on the same plate. * *p* < 0.05, *** *p* < 0.001 for relevant groups.

It was possible that phototherapy killed both host cells and intracellular amastigotes. Therefore, the effect of light treatment on the viability of uninfected macrophages was determined. Light treatment had a dose-dependent effect on macrophage viability, with the lowest dose having no significant effect on macrophage viability in two of the four experiments (mean % suppression ± SD for four separate experiments, 13 ± 17). Treatment at 66 J/cm^2^ caused significantly greater suppression in the viability of uninfected macrophages compared with treatment at 33 J/cm^2^ (Figure 5). However, this higher dose did not cause a significantly greater reduction in *L. major* or *L. mexicana* parasite burdens, perhaps indicating that light treatment was more toxic to uninfected macrophages compared with *Leishmania*-infected macrophages.

### 3.3. Photo-Treatment Using 45 J/cm^2^ per Day Had No Consistent Suppressive Effect on the In Vivo Growth of L. major

**Figure 6:**
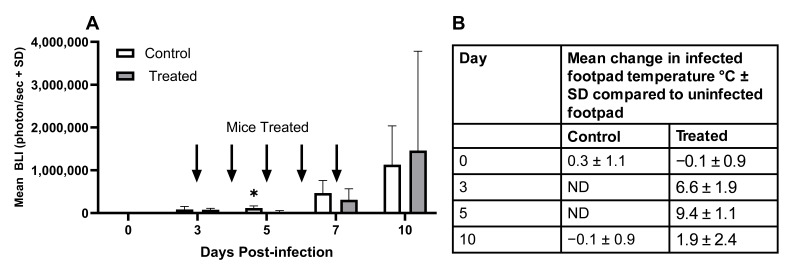
The effect of 405 nm photo-treatment on the parasite burden of *L. major*-infected mice (**A**) and the change in footpad temperature (**B**). *L. major*-infected mice were given local light treatment (45 J/cm^2^) on the infected footpad, once/day for 5 days starting on day 3 post-infection. Parasite burdens in control and treated mice (*n* = 5/treatment) were assessed over the course of the experiment using IVIS imaging (**A**). The effect of light treatment on footpad temperature was determined by comparing the difference in the temperature of the uninfected and infected footpad in infected mice (**B**). * *p* < 0.05 compared to controls.

The ability of light treatment to significantly reduce *Leishmania* *in vivo* parasite growth was assessed using a murine model of cutaneous leishmaniasis, which can be used to monitor parasite growth in the footpad from day 3 post-infection using IVIS imaging. This is a mild disease model, and animals were closely monitored during treatment for any sign of host cytotoxicity. Mice were treated individually on days 3–7 using the custom-built apparatus shown in Figure 1 to deliver 45 J/cm^2^ each day to the site of infection. Figure 6A shows that treatment was associated with a significant reduction in parasite burdens on day 5 post-infection (*p* < 0.05). By day 7, parasite burdens had increased and were similar in treated and control groups, and on day 10, there was no significant difference in the mean size of the infected footpad in control and treated mice (mean difference in footpad size for uninfected and infected footpad ± SD [mm], control 0.45 ± 0.16, treated 0.63 ± 0.87). Treatment was associated with an increase in footpad temperature (Figure 6B), but in all cases, the footpad temperature reading was below 37 °C. In a repeat experiment, parasite growth in control and light-treated mice was similar over the course of the study and the transient decrease in parasite growth on day 5 observed in IVIS imaging studies did not occur. A preliminary study showed that heat alone was not responsible for the transient reduction in parasite growth observed in the first experiment, as heating the infected footpad for the same treatment time at 37 °C had no significant effect on parasite survival over the course of the study based on IVIS imaging, and the mean increase in footpad size in control and heat-treated *L. major*-infected mice by day 10 post-infection was similar for the two groups (control 0.30 ± 0.17, heat-treated 0.22 ± 0.10). Images from the two *in vivo* IVIS studies are shown in the Supplementary Data (Figures S3 and S4).

## 4. Discussion

The results of this study showed that 405 nm violet-blue light treatment successfully reduced parasite numbers *in vitro*, demonstrating a dose-dependent effect on the survival of extracellular promastigotes and intracellular amastigotes of *L. major* and *L. mexicana.* This is perhaps not surprising, as previous studies have shown the susceptibility of a range of other microorganisms to light of this wavelength [10] and more recently that *L. donovani* promastigotes, present in spiked platelets, were killed by violet-blue light [15]. Unfortunately, we could not demonstrate that blue light caused consistent killing *in vivo* using a relatively mild *L. major* footpad infection model. Our studies using uninfected macrophages showed that light treatment was consistently toxic to uninfected macrophages at doses above 66 J/cm^2^. Therefore, it is important to focus the light beam on infected tissue to minimize unwanted cytotoxic effects to host cells. Our mild disease model could detect the presence of *L. major* amastigotes within the footpad from day 3 post-infection. Therefore, starting treatment on day 3 post-infection would have allowed light treatment to stop parasite growth whilst burdens were at the lowest detectable level. We gave one treatment/day for 5 days, as this seems a reasonable protocol for people to self-treat. In future studies, a more severe traditional rump lesion animal model would be justified, as it would be comparable with the type of lesions presented in the clinic. Presumably, the ability of the light to access deep into the skin is a problem. One way to improve the efficacy of the light treatment would be to apply a topical light sensitizer. A study determining the efficacy of light treatment on cancer growth showed that using the photosensitizer Radagel^®^ increased the ability of light at 405 nm to penetrate skin tissues [20]. Another study using a murine cutaneous *Aspergillus fumigatus* infection model found that treatment with quinine hydrochloride increased the antifungal efficacy of antimicrobial blue light. In this model, fungal burdens were determined using IVIS imaging [21]. A single light dose of 576 J/cm^2^ (20 mW/cm^2^ irradiance for 80 min) caused a 1.85 log_10_ RLU lower than the PBS control (*p* = 0.028), whereas treatment with light and quinine hydrochloride caused a 2.09 log_10_ decrease in RLU compared to PBS controls (*p* = 0.028). In these studies, the light dose was given by attaching a collimator to the infected site and the irradiance dose was adjusted through manipulating the distance between the LED aperture and the target using a PM100D power meter. Wearable light treatment devices which can fit skin surfaces in different body areas are being designed [22], but at present, nothing to deliver light at 405 nm is available. Increasing the light dose does present some problems, as this would either require a longer treatment time or an increase in the irradiation dose administered. There is a maximum time that animals can be under an anesthetic for their well-being, based on how many times this is used and the length of time that animals are under anesthesia. An injectable anesthetic could be used in future studies, but care would have to be taken so that the infection site does not get too hot and cause a skin burn. This risk could be minimized by using a different LED array, using a photoinitator to increase the efficacy of LED treatment [23], and/or using a cooling system to minimize the heat generated by the light source [24]. Unfortunately, animal studies rely on behavioral cues or temperature measurements to monitor well-being. It would be possible to get direct feedback on any adverse effect(s) caused by light treatment in human studies. In a recent study, a significant reduction in parasite burdens was obtained in *L. amzonenesis*-infected mice but a distinctly different experimental arrangement was used. In the study of Pimenta et al. [16], *L. amazonensis*-infected mice with a lesion in the dorsal area were exposed to blue LED phototherapy (7 μW/cm^2^) for 12 h per day over 10 days. In contrast, the present study utilized higher irradiance light (0.15 W/cm^2^) applied directly to the infected footpad for short 5 min treatment times. This would mean that in the Pimenta et al. study [16], the mice were exposed to a light dose of 3.024 J/cm^2^, which is significantly lower than the dose used in the study reported here (45 J/cm^2^). However, it is likely that the dose administered to the lesion in Pimenta’s study was even lower, as the light treatment was not directly applied to the lesion site as mice would be moving around the cage during the 12 h treatment period (although this is not detailed in the paper). Further investigations would need to be conducted to evaluate the comparative efficacy of continuous versus intermittent treatments for clinical application. Data from recent *in vitro* antibacterial studies has demonstrated the improved germicidal efficacy of low- versus high-irradiance light treatments [25] and, therefore, further work investigating the influence of the light irradiances used will be important. Additionally, there is potential that exposure of the whole mouse to light treatment results in a general systemic activation which helps to clear parasites from the host. It is well known that the resistance of certain mouse strains to leishmanial infection can be related to their macrophages having a significantly higher inherent production of nitric oxide compared to macrophages from susceptible mouse strains [26]. Thus, one of our prime aims was to use a treatment regimen that could be used in clinical studies. In the Pimenta et al. study [16], mice were treated on day 60 post-infection, where the skin barrier would have been compromised by the development of a lesion. This may have allowed better light access to parasites within the infected dorsal area. However, in this study, we used a protocol that allowed detection of parasites within the footpad without the development of a footpad lesion to minimize discomfort in the infected mice. Different *Leishmania* spp. were used in the two studies, which may vary in their susceptibility to 405 nm light treatment. Indeed, there was some evidence in the present study that intracellular *L. mexicana* parasites were less susceptible than *L. major* parasites to light treatment. Unfortunately, it is not possible to directly compare the *in vitro* results for the different *Leishmania* species used in the two studies, as the irradiance used for *in vitro* studies is not stated for Pimenta’s study. However, our study provides evidence that light at 405 nm is directly toxic to two additional *Leishmania* species.

The results of the present study show that the complexity of the medium at treatment had a significant effect on therapeutic outcome, as there was significantly less promstigote parasite killing (*p* < 0.001) when light treatment was carried out in saline rather than complete medium. This was not unexpected, as cell culture medium is known to contain photosensitive components which can become photoexcited upon exposure to violet-blue light, causing the production of reactive oxygen intermediates (ROS) which can amplify the antimicrobial effects of the light treatment alone. ROS are known to be directly toxic to *Leishmania* parasites, but their contribution to parasite killing can depend on the leishmanial species and the stage of infection [27]. A recent study using an H_2_DCFDA assay showed that ROS were produced by platelets spiked with *L. donovani* promastigotes treated with violet-blue light and ROS production was associated with parasite killing [15].

The data from the present study indicated that *Leishmania*-infected macrophages were more resistant to killing from exposure to light at 405 nm than uninfected macrophages, and that *L. mexicana* amastigotes were more resistant to killing than intracellular *L. major* amastigotes or *L. major* and *L. mexicana* promastigotes. This is perhaps not surprising, as studies have shown that *Leishmania*-infected cells are more resistant to apoptosis by various mechanisms, including a reduction in the amount of cytochrome C present within cells [28]. In addition, *Leishmania*-infected macrophages inherently produce lower amounts of ROS than uninfected macrophages on stimulation, as the parasites upregulate antioxidant mechanisms in infected cells [29]. *L. mexicana* amastigotes live within megasomes, which are large lysosome-like structures [30], which may inhibit antiparasitic ROS access. A high level of killing of *L. major* and *L. mexicana* promastigotes and amastigote parasites (>93%) occurred at a dose of 101.2 J/cm^2^. This is comparable to levels reported for other microorganisms, for example, a dose of 108 J/cm^2^ killed 98% *S. aureus* bacteria [31], 93% inactivation of *Salmonella enterica typhimurium* occurred at a dose of 110 J/cm^2^, and 100% killing occurred at a dose of 128 J/cm^2^ for *L. monocytogenes* [32]. The challenge will be achieving this level of exposure within the cutaneous tissues for CL treatment, whilst minimizing damage to healthy tissue, as the present study demonstrated that light treatment is cytotoxic to mammalian macrophages.

The present study, therefore, clearly demonstrates that light at 405 nm is toxic to both *L. major* and *L. mexicana* promastigote and intracellular amastigote parasites *in vitro*, but this does not clearly translate to an *in vivo* effect using our protocol. It also demonstrates that intracellular amastigotes are more resistant to killing than extracellular promastigotes. However, further studies are required to identify a treatment regimen for *in vivo* infections which could safely be used for clinical treatment of cutaneous leishmaniasis.

## Data Availability

The original contributions presented in this study are included in the article/Supplementary Material. Further inquiries can be directed to the corresponding author.

## Supplementary Materials

The following supporting information can be downloaded at: https://www.mdpi.com/article/10.3390/microorganisms14051135/s1, Figure S1: A photo of the type of data obtained in *in vitro* studies. Figure S2: An example of the type of images obtained in IVIS imaging studies to show the region of interest (ROI) used in studies. Figure S3: The effect of light treatment on the parasite burdens of *L. major* infected mice. Figure S4: The effect of light treatment and heat treatment on the parasite burdens of *L. major* infected mice.

## References

[1] (2023). World Health Organization Factsheets. Leishmaniasis. https://www.who.int/news-room/fact-sheets/detail/leishmaniasis.

[2] Pearson R.D., de Queiroz Sousa A. (1996). Clinical Spectrum of Leishmaniasis. Clin. Infect. Dis..

[3] Blackwell J.M., Fakiola M., Castellucci L.C. (2020). Human genetics of leishmania infections. Hum. Genet..

[4] Herwaldt B.L. (1999). Leishmaniasis. Lancet.

[5] Bennis I., De Brouwere V., Belrhiti Z. (2018). Psychosocial burden of localised cutaneous Leishmaniasis: A scoping review. BMC Public Health.

[6] Nascimento E., Fernandes D.F., Vieira E.P., Campos-Neto A., Ashman J.A., Alves F.P., Coler R.N., Bogatzki L.Y., Kahn S.J., Beckmann A.M. (2010). A clinical trial to evaluate the safety and immunogenicity of the LEISH-F1+MPL-SE vaccine when used in combination with meglumine antimoniate for the treatment of cutaneous leishmaniasis. Vaccine.

[7] Younis B.M., Osman M., Khalil E.A.G., Santoro F., Furini S., Wiggins R., Keding A., Carraro M., Musa A.E.A., Abdarahaman M.A.A. (2021). Safety and immunogenicity of ChAd63-KH vaccine in post-kala-azar dermal leishmaniasis patients in Sudan. Mol. Ther..

[8] Orabi M.A.A., Lahiq A.A., Awadh A.A.A., Alshahrani M.M., Abdel-Wahab B.A., Abdel-Sattar E.S. (2023). Alternative Non-Drug Treatment Options of the Most Neglected Parasitic Disease Cutaneous Leishmaniasis: A Narrative Review. Trop. Med. Infect. Dis..

[9] Shmueli M., Ben-Shimol S. (2024). Review of Leishmaniasis Treatment: Can We See the Forest through the Trees?. Pharmacy.

[10] Tomb R.M., White T.A., Coia J.E., Anderson J.G., MacGregor S.J., Maclean M. (2018). Review of the Comparative Susceptibility of Microbial Species to Photoinactivation Using 380–480 nm Violet-Blue Light. Photochem. Photobiol..

[11] Ramakrishnan P., Maclean M., MacGregor S.J., Anderson J.G., Grant M.H. (2014). Differential sensitivity of osteoblasts and bacterial pathogens to 405 nm light highlighting potential for decontamination applications in orthopaedic surgery. J. Biomed. Opt..

[12] Maclean M., MacGregor S.J., Anderson J.G., Woolsey G.A. (2008). The Role of Oxygen in the Visible-Light Inactivation of *Staphylococcus aureus*. J. Photochem. Photobiol. B Biol..

[13] Maclean M., Anderson J.G., MacGregor S.J., White T., Atreya C.D. (2016). A New Proof of Concept in Bacterial Reduction: Antimicrobial Action of Violet-Blue Light (405 nm) in Ex Vivo Stored Plasma. J. Blood Transfus..

[14] Ramakrishnan P., Maclean M., MacGregor S.J., Anderson J.G., Grant M.H. (2016). Cytotoxic responses to 405 nm light exposure in mammalian and bacterial cells: Involvement of reactive oxygen species. Toxicol. Vitr..

[15] Kaldhone P.R., Azodi N., Markle H.L., Dahiya N., Stewart C., Anderson J., MacGregor S., Maclean M., Nakhasi H.L., Gannavaram S. (2024). The Preclinical Validation of 405 nm Light Parasiticidal Efficacy on *Leishmania donovani* in Ex Vivo Platelets in a Rag2^−/−^ Mouse Model. Microorganisms.

[16] Pimenta B.L., Lage D.P., de Freitas C.S., Vale D.L., Falcão K.O.M., Dias S.S.G., Câmara R.S.B., Pereira I.A.G., Silva A.L., Duarte Júnior L.A. (2025). Blue light-emitting diode phototherapy presents in vitro efficacy against distinct *Leishmania* species and is therapeutic against tegumentary leishmaniasis in BALB/c mice. Front. Immunol..

[17] Carter K.C., Hutchison S., Boitelle A., Murray H.W., Sundar S., Mullen A.B. (2005). Sodium stibogluconate resistance in *Leishmania donovani* correlates with greater tolerance to macrophage antileishmanial responses and trivalent antimony therapy. Parasitology.

[18] Rampersad S.N. (2012). Multiple applications of Alamar Blue as an indicator of metabolic function and cellular health in cell viability bioassays. Sensors.

[19] Alsaadi M., Italia J.L., Mullen A.B., Ravi Kumar M.N., Candlish A.A., Williams R.A., Shaw C.D., Al Gawhari F., Coombs G.H., Wiese M. (2012). The efficacy of aerosol treatment with non-ionic surfactant vesicles containing amphotericin B in rodent models of leishmaniasis and pulmonary aspergillosis infection. J. Control. Release.

[20] Shakhova M., Elagin V., Plekhanov A., Khilov A., Kurakina D., Kamensky V., Kirillin M. (2024). Post-Operational Photodynamic Therapy of the Tumor Bed: Comparative Analysis for Cold Knife and Laser Scalpel Resection. Biomedicines.

[21] Leanse L.G., Dos Anjos C., Wang Y., Murray C.K., Hooper D.C., Dai T. (2021). Effective Treatment of Cutaneous Mold Infections by Antimicrobial Blue Light That Is Potentiated by Quinine. J. Infect. Dis..

[22] Cho E.H., Kim Y.W., Sim J., Yeon H., Baek S., Jeong S.M., Lee J., Jeon Y., Choi K.C. (2025). Recent advances in flexible and wearable OLEDs for biomedical applications: A review. Mater. Horiz..

[23] Liu S., Borjigin T., Schmitt M., Morlet-Savary F., Xiao P., Lalevée J. (2023). High-Performance Photoinitiating Systems for LED-Induced Photopolymerization. Polymers.

[24] Ong J., Nazarian A., Tam J., Farinelli W., Korupolu S., Drake L., Isaacson B., Pasquina P., Williams D. (2023). An antimicrobial blue light device to manage infection at the skin-implant interface of percutaneous osseointegrated implants. PLoS ONE.

[25] Sinclair L.G., Anderson J.G., MacGregor S.J., Maclean M. (2024). Enhanced antimicrobial efficacy and energy efficiency of low irradiance 405-nm light for bacterial decontamination. Arch. Microbiol..

[26] Liew F.Y., Li Y., Moss D., Parkinson C., Rogers M.V., Moncada S. (1991). Resistance to *Leishmania major* infection correlates with the induction of nitric oxide synthase in murine macrophages. Eur. J. Immunol..

[27] Roy S., Mandal M., Halder M., Das P.K., Ukil A. (2025). Oxidative Stress and Survival of *Leishmania* spp.: A Relationship of Inverse Proportionality for Disease Outcome. Expert Rev. Mol. Med..

[28] Fernandes J.C.R., Zamboni D.S. (2024). Mechanisms regulating host cell death during *Leishmania* infection. mBio.

[29] Carneiro P.P., Conceição J., Macedo M., Magalhães V., Carvalho E.M., Bacellar O. (2016). The Role of Nitric Oxide and Reactive Oxygen Species in the Killing of *Leishmania braziliensis* by Monocytes from Patients with Cutaneous Leishmaniasis. PLoS ONE.

[30] Alexander J., Vickerman K. (1975). Fusion of host cell secondary lysosomes with the parasitophorous vacuoles of *Leishmania* mexicana-infected macrophages. J. Protozool..

[31] Tomb R.M., Maclean M., Coia J.E., MacGregor S.J., Anderson J.G. (2017). Assessment of the potential for resistance to antimicrobial violet-blue light in *Staphylococcus aureus*. Antimicrob. Resist. Infect. Control.

[32] Wang Y., Wang Y., Wang Y., Murray C.K., Hamblin M.R., Hooper D.C., Dai T. (2017). Antimicrobial blue light inactivation of pathogenic microbes: State of the art. Drug Resist. Updat..

