# Supplementary material for: Light Photo Treatment at 405 nm Can Effectively Kill Leishmania Parasites
Source: Microorganisms. 2026 May 16;14(5):1135. doi: 10.3390/microorganisms14051135 (PMC13209302; doi:10.3390/microorganisms14051135)

Supplemetary data for the manuscript 405nm light photo treatment can effectively kill *Leishmania* parasites

Figure S1 A photo of the type of data obtained in *in vitro* studies. Page 1

Figure S2 An example of the type of images obtained in IVIS imaging studies to show the region of interest (ROI) used in studies. Page 3

Figure S3 The effect of light treatment on the parasite burdens of *L. major* infected mice. Page 4

Figure S4 The effect of light treatment and heat treatment on the parasite burdens of *L. major* infected mice. Page 8

Figure S1 A photo of the type of data obtained in *in vitro* studies. Luciferin stock solution (20 µl in PBS pH 7.4, final concentration 150 µg/ml) was added to each sample at the end of the incubation period and the bioluminescent signal (medium binning) emitted by each sample was determined using the 8 × 12 grid provided by the system software and automatic setting. The data was exported into to excel and used to determine the effect of light treatment on parasite survival.

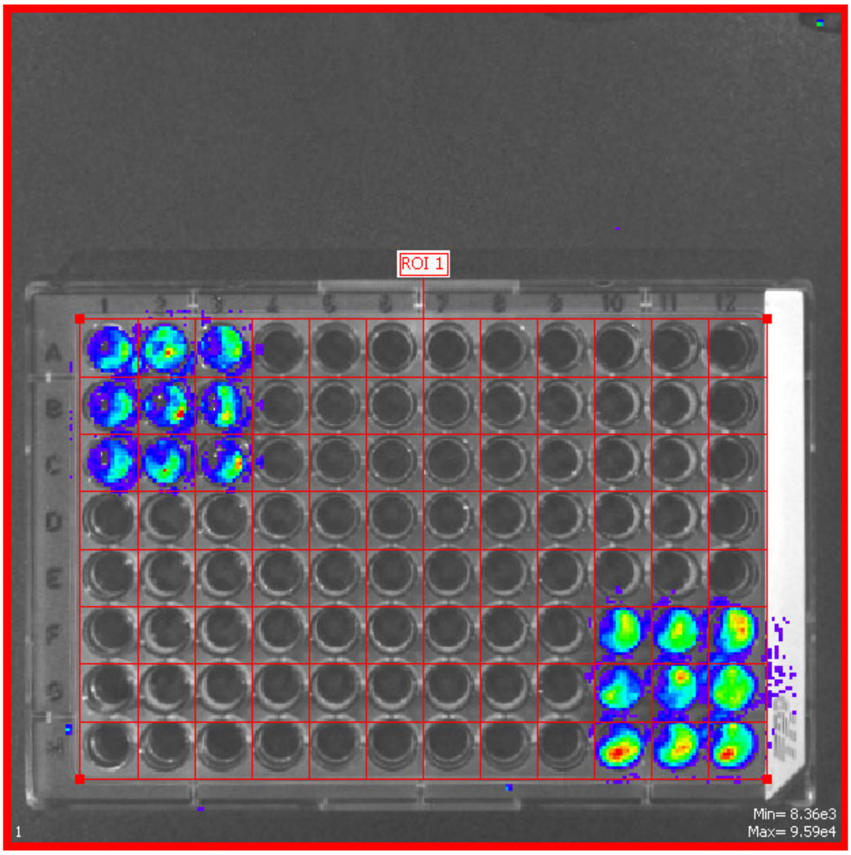

Figure S2 An example of the type of images obtained in IVIS imaging studies to show the region of interest (ROI) used in studies. The bioluminescent signal emitted by the uninfected and infected footpad of mouse was determined 5 min after intraperitoneal injection of luciferin solution (150 mg/kg, medium binning, 2-minute imaging time). The same sized region of interest was used for the footpad of each mouse at each time point in an experiment. The amount of bioluminescence (BLI) emitted in each region of interest (ROI) was determined using the Living Image software, and the results were recorded as photons/sec emitted. The parasite-specific bioluminescent signal for each infected mouse was determined by subtracting the bioluminescent signal of the uninfected footpad from the signal emitted by the infected footpad for each mouse. Two images over a 2 minute time peiod were taken for each group of mice. The data was exported into to excel and used to determine the effect of light treatment on parasite survival.

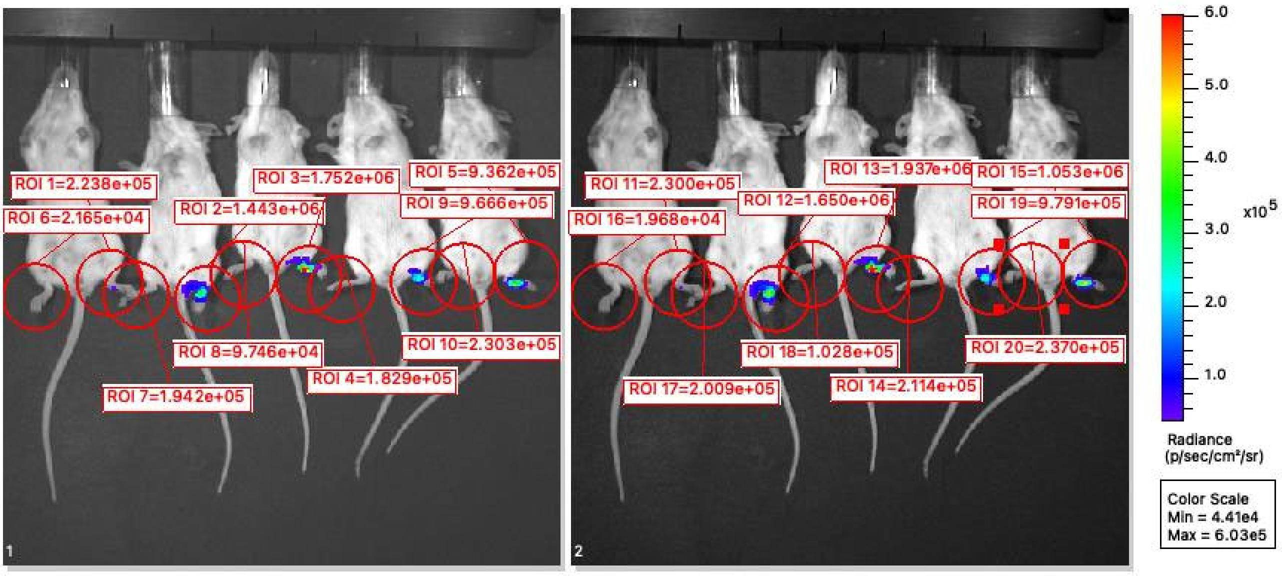

Figure S3 The effect of light treatment on the parasite burdens of *L. major* infected mice. BALB/c female mice infected with *L. major* luciferase expressing promastigotes were treated once/day for 5 minutes on days 3-8 with a dose of 0.15 W/cm^2 to give a dose of 45 J/cm^2/day. The effect of treatment on parasite growth was determined by comparing the bioluminescent signal emitted by the uninfected and infected footpad of each control and treated mouse 5 min after intraperitoneal injection of luciferin solution (150 mg/kg, medium binning, 2-minute imaging time). The same sized region of interest was used for the footpad of each mouse at each time point. Images were taken on pretreament on day 3 post-infection and on days 5 and 10 post-infection.

Day 3 Pretreatment Control Group

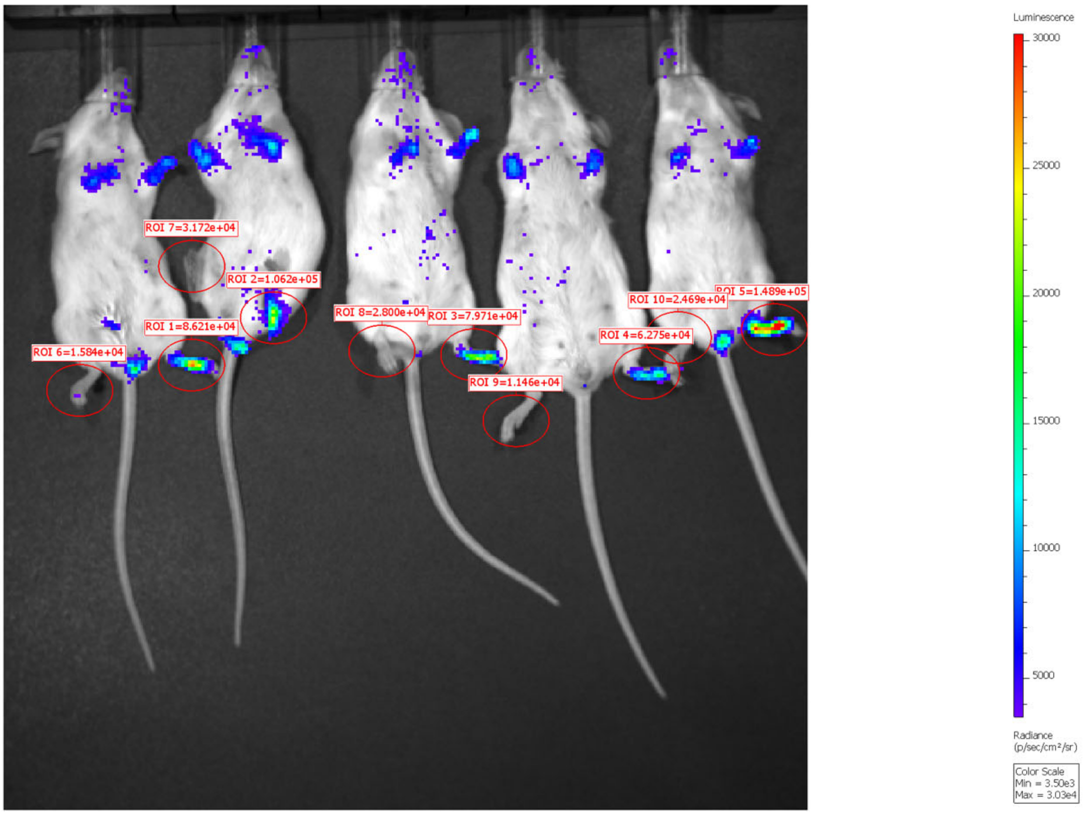

Day 3 Pretreatment treated group

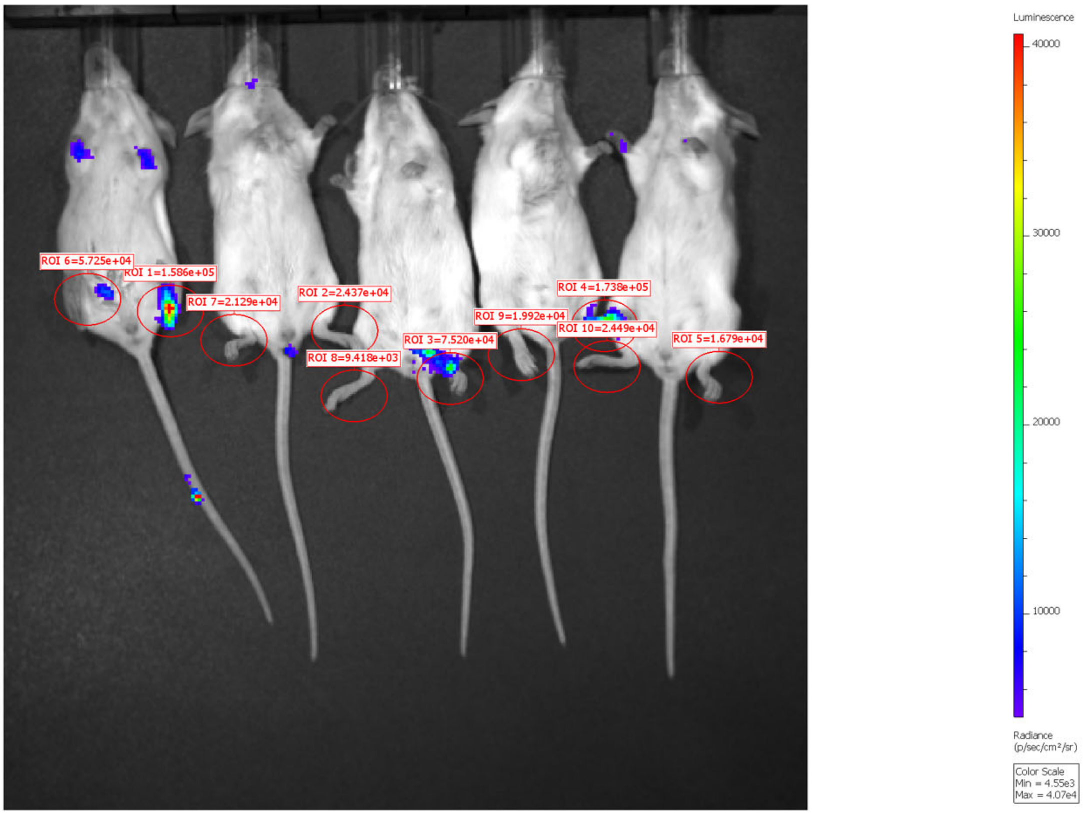

Day 5 control group

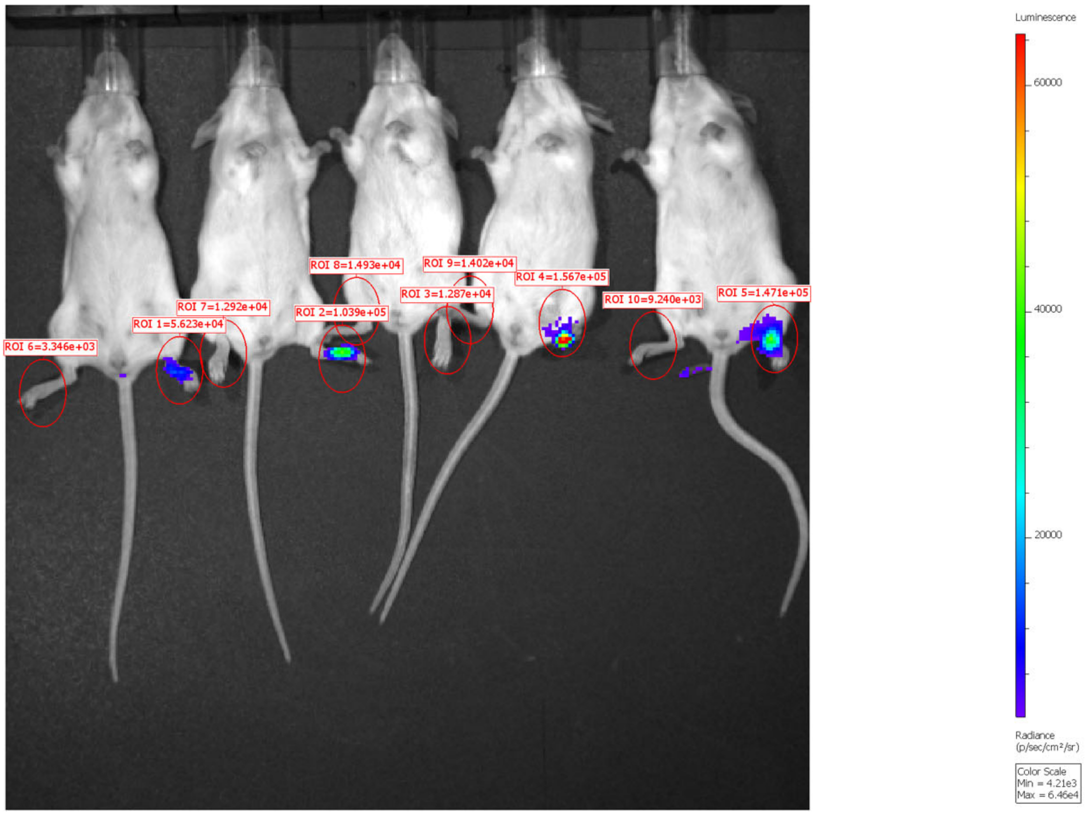

Day 5 treated group

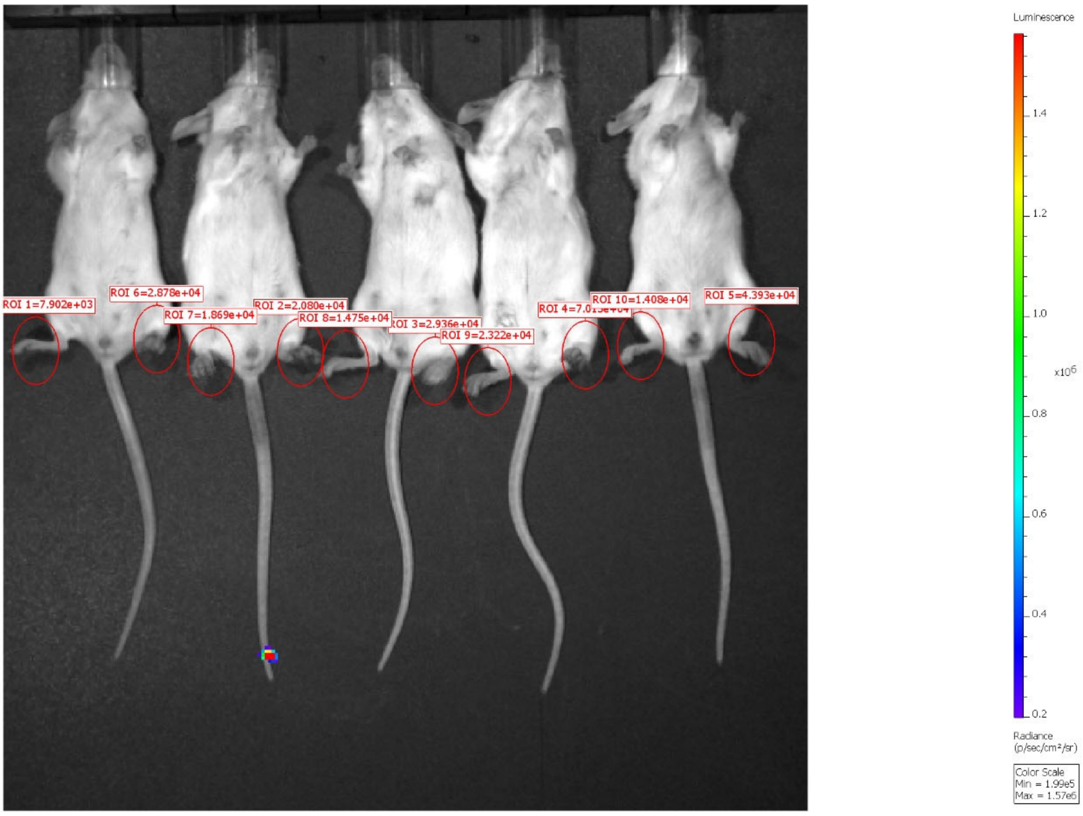

Day 10 control group

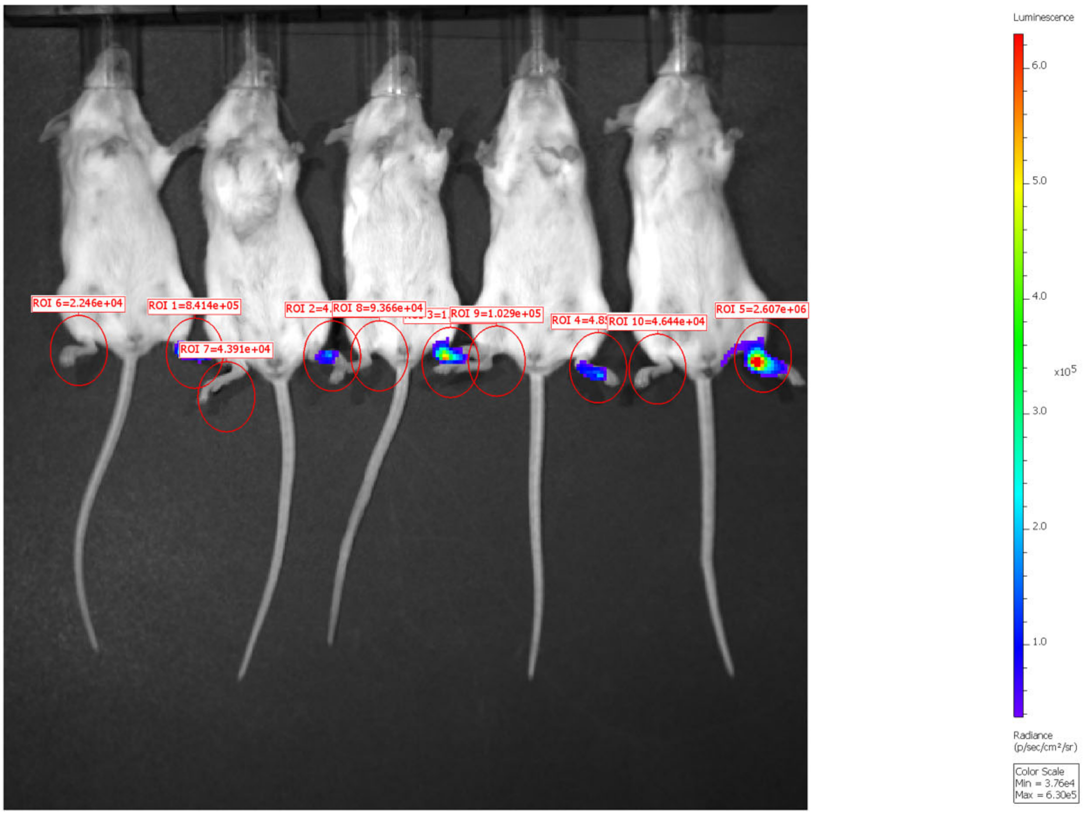

Day 10 treated group

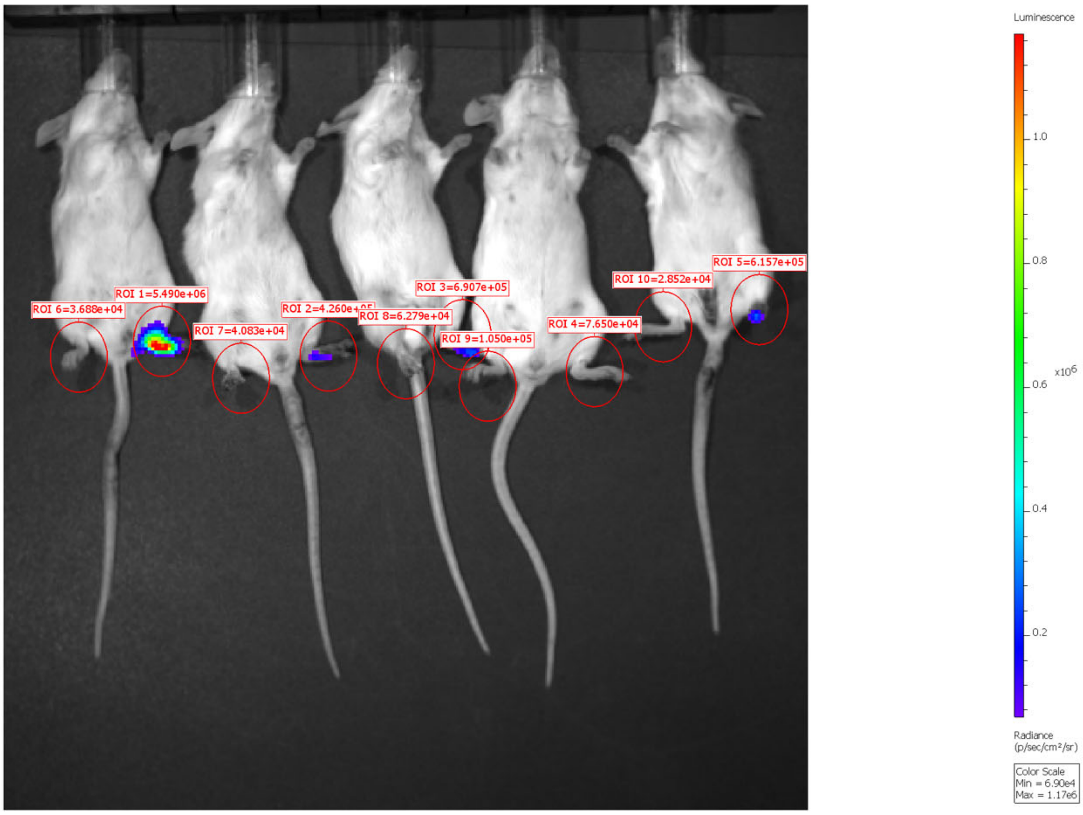

Figure S4 The effect of light treatment and heat treatment on the parasite burdens of *L. major* infected mice. BALB/c female mice infected with *L. major* luciferase expressing promastigotes were treated once/day for 5 minutes on days 3-8 with a dose of 0.15 W/cm^2 to give a dose of 45 J/cm^2/day. In addition the effect of heating the infected footpad to 37°C on parasite survival was determined by placing the infected footpad of an anaesthetized mouse (inhaled anesthesia) in a water bath at 37°C for 5 minutes/day on days 3-7 post-infection to mimic the effects of the heat generated by light treatment. The effect of treatment on parasite growth was determined by comparing the bioluminescent signal emitted by the uninfected and infected footpad of each control and treated mouse 5 min after intraperitoneal injection of luciferin solution (150 mg/kg, medium binning, 2-minute imaging time). The same sized region of interest was used for the footpad of each mouse at each time point. Images were taken on pretreament on day 3 post-infection and on days 6 and 10 post-infection.

Day 3 control group

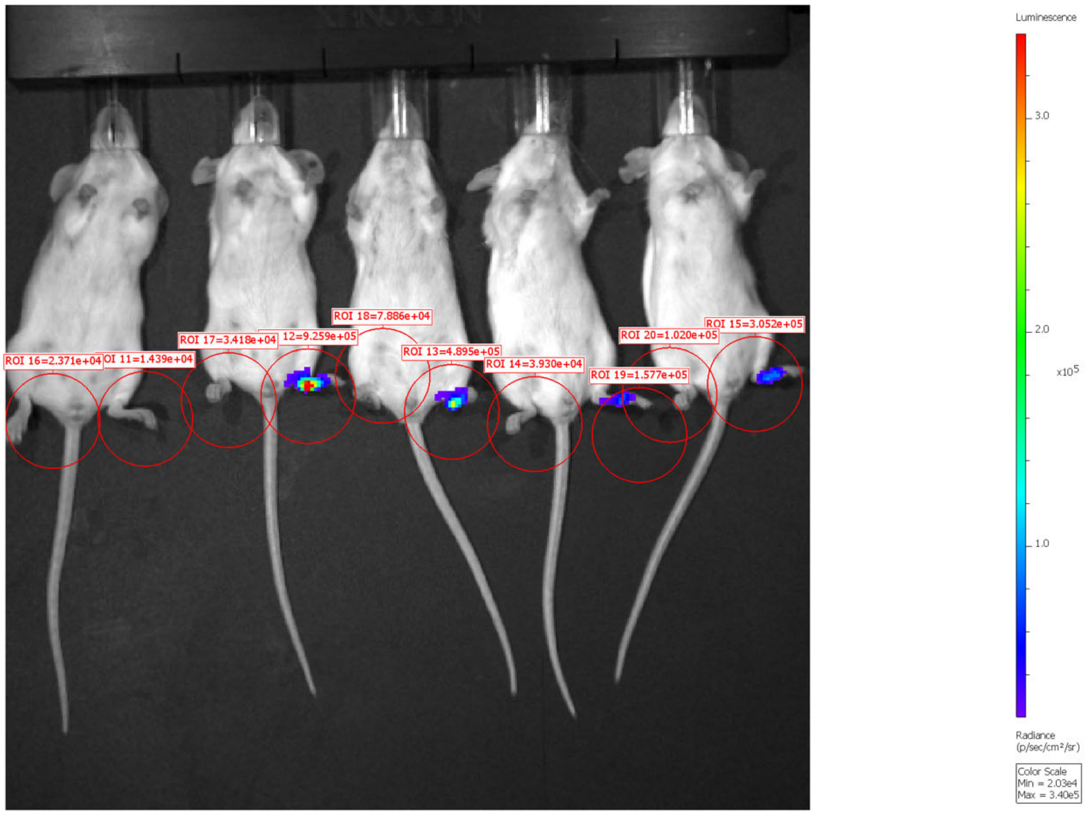

Day 3 light treated group

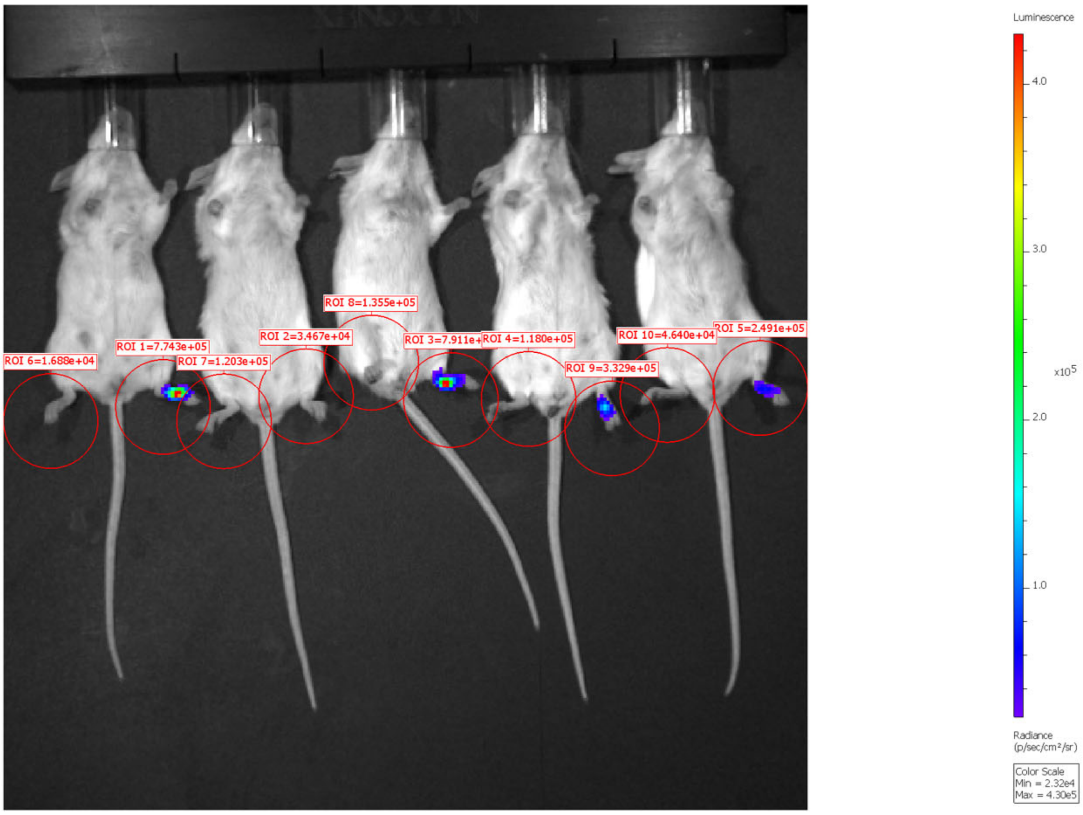

Day 3 heat treated group

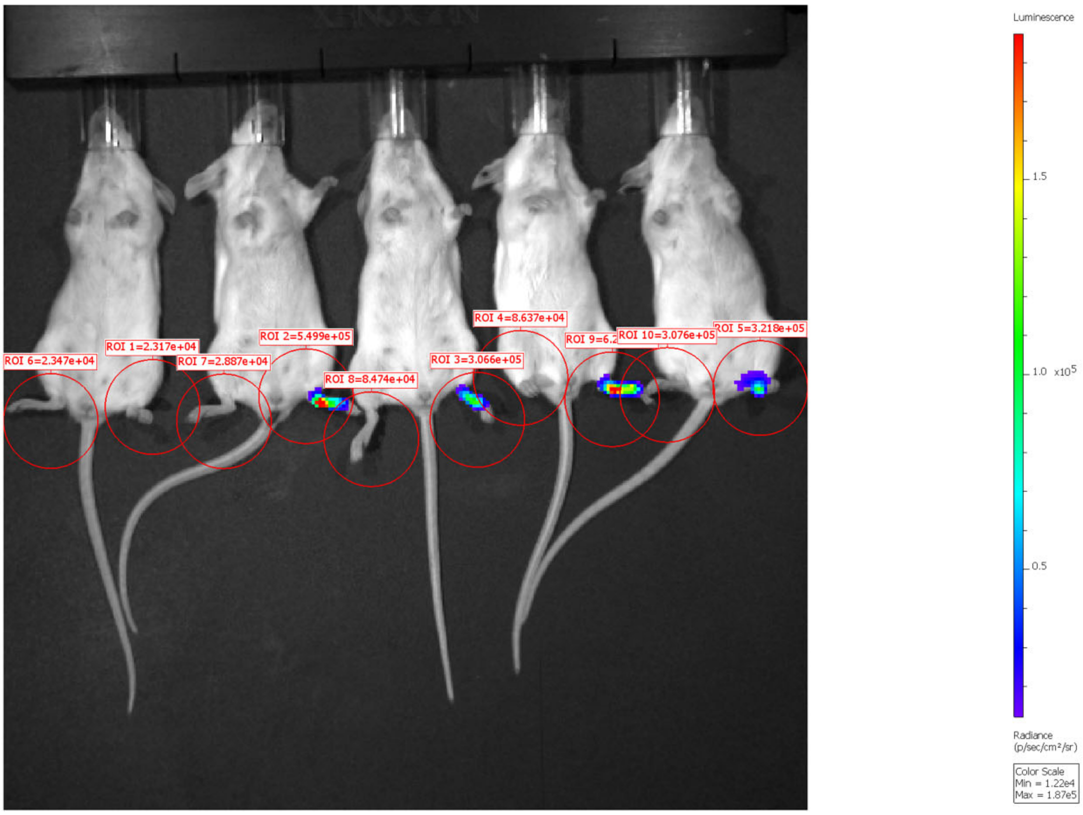

Day 6 Control group

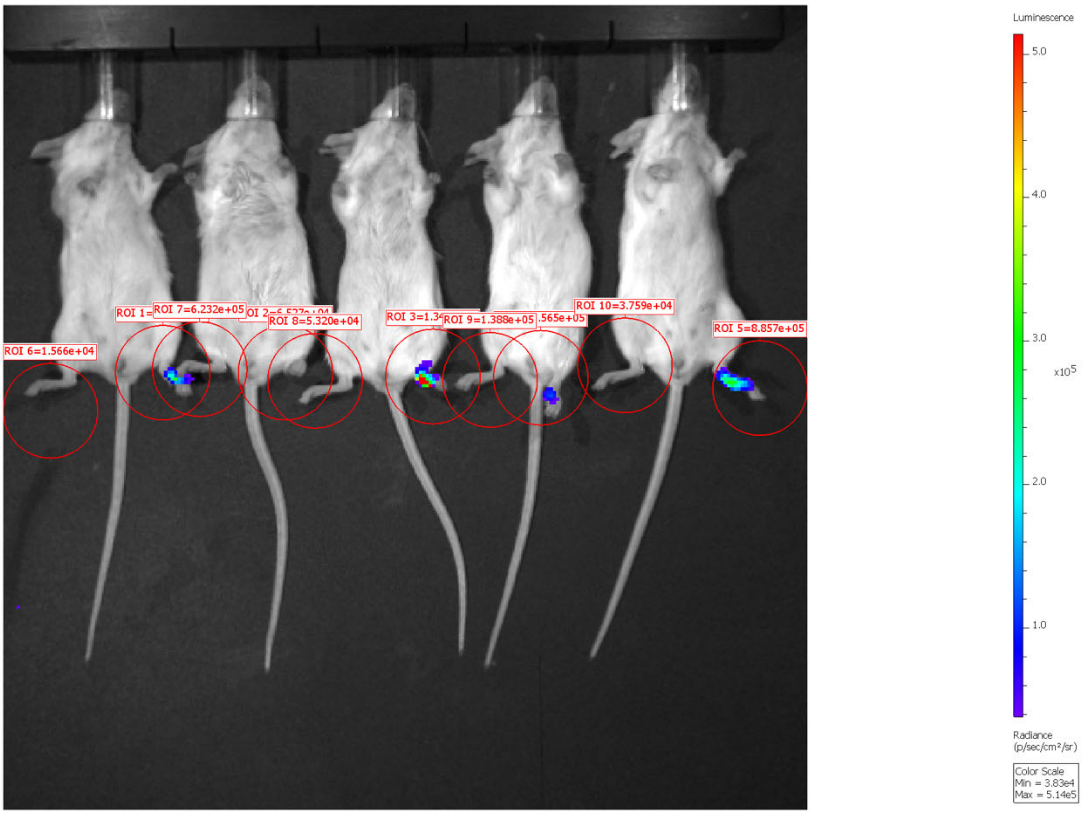

Day 6 light treated group

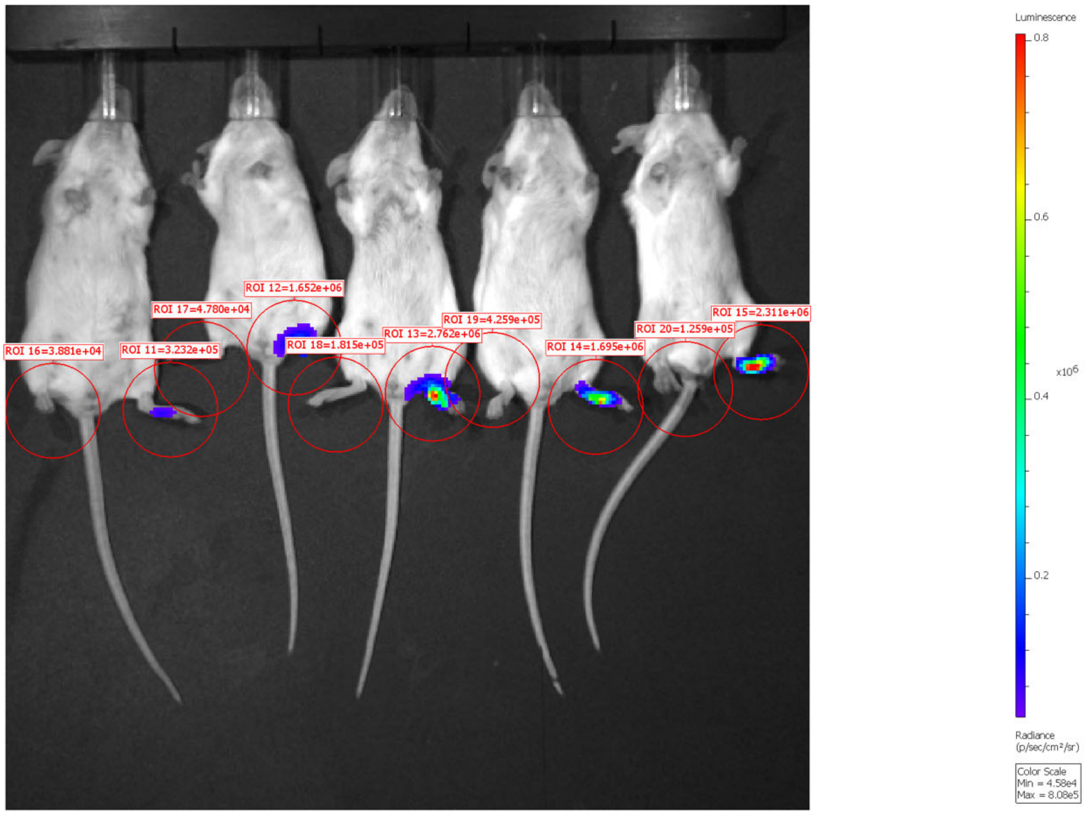

Day 6 Heat treated group

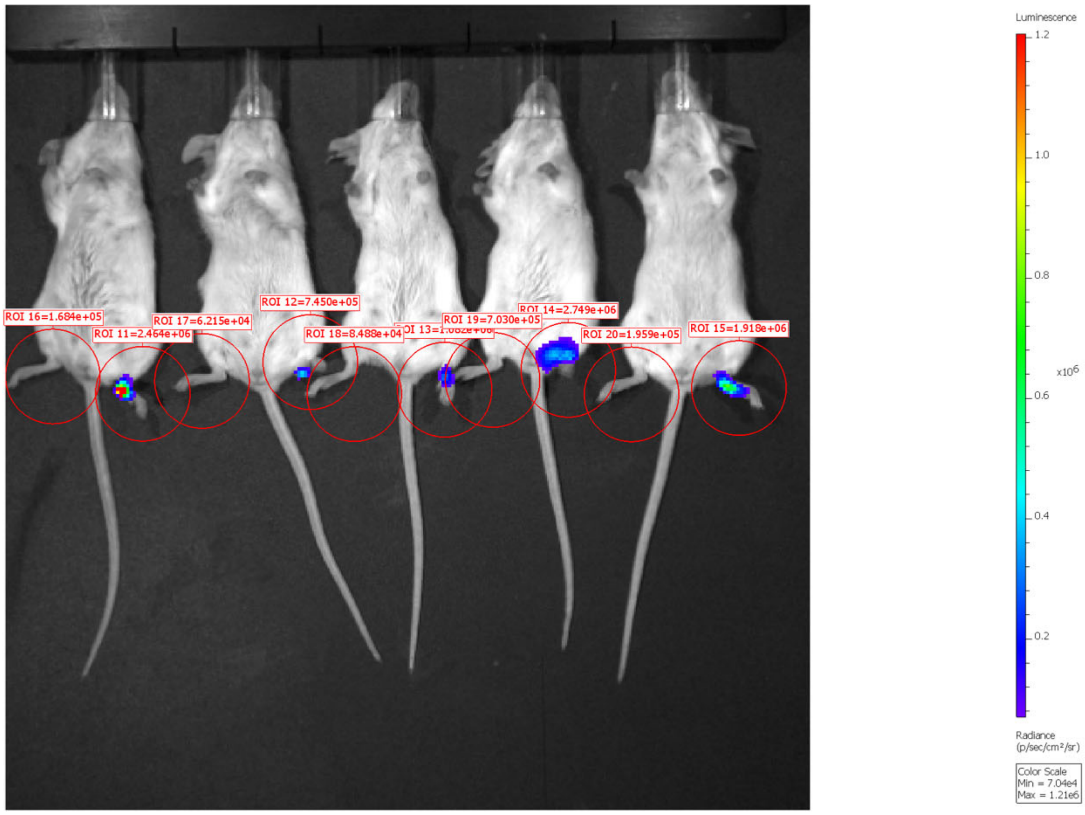

Day 10 Control group

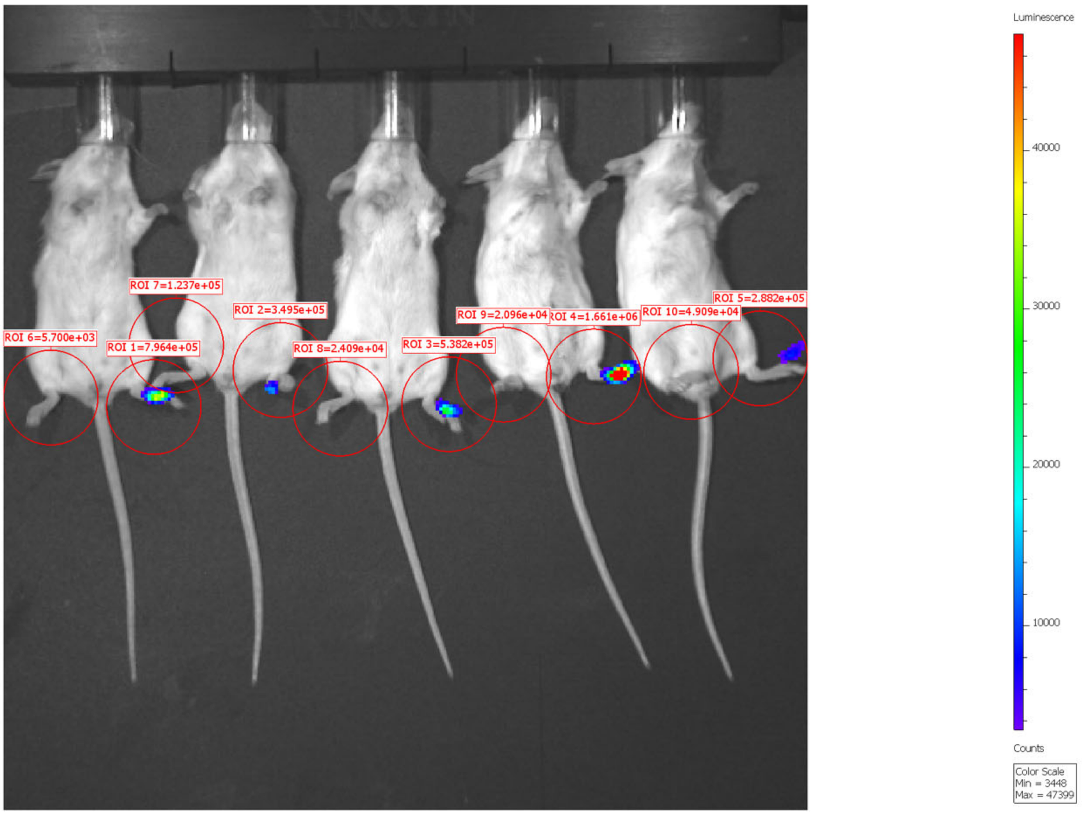

Day 10 Light treated group

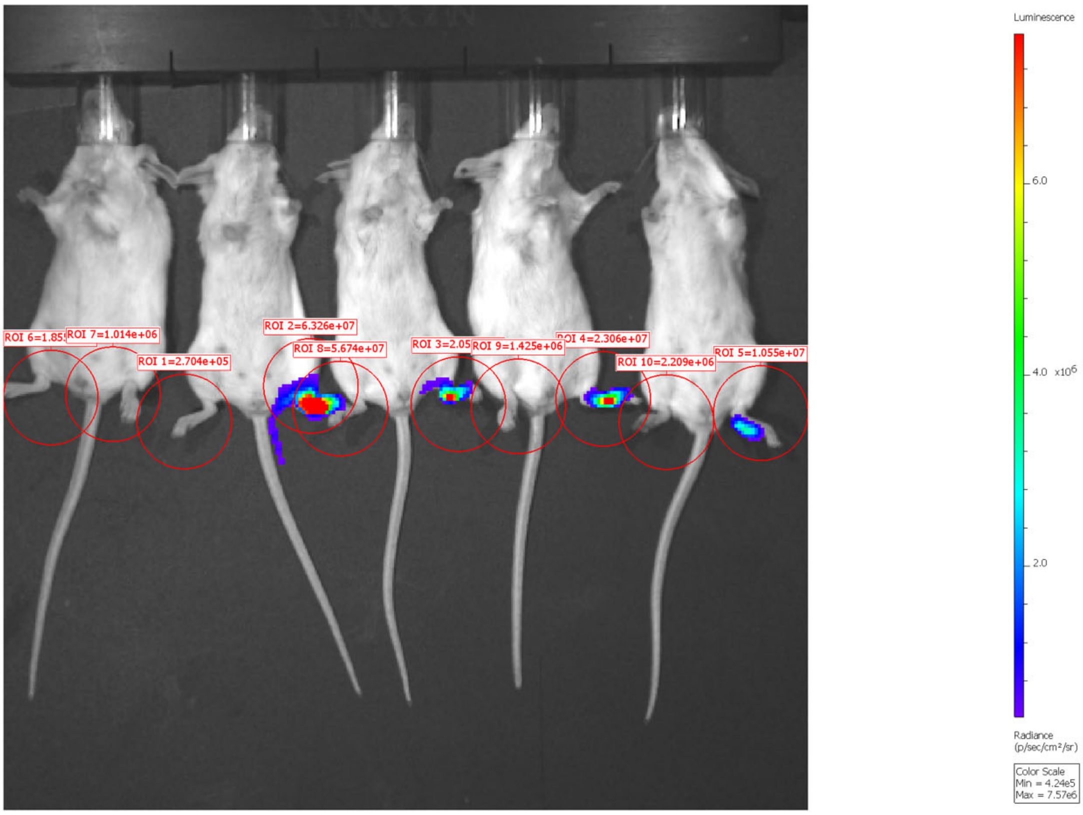

Day 10 Heat treated group

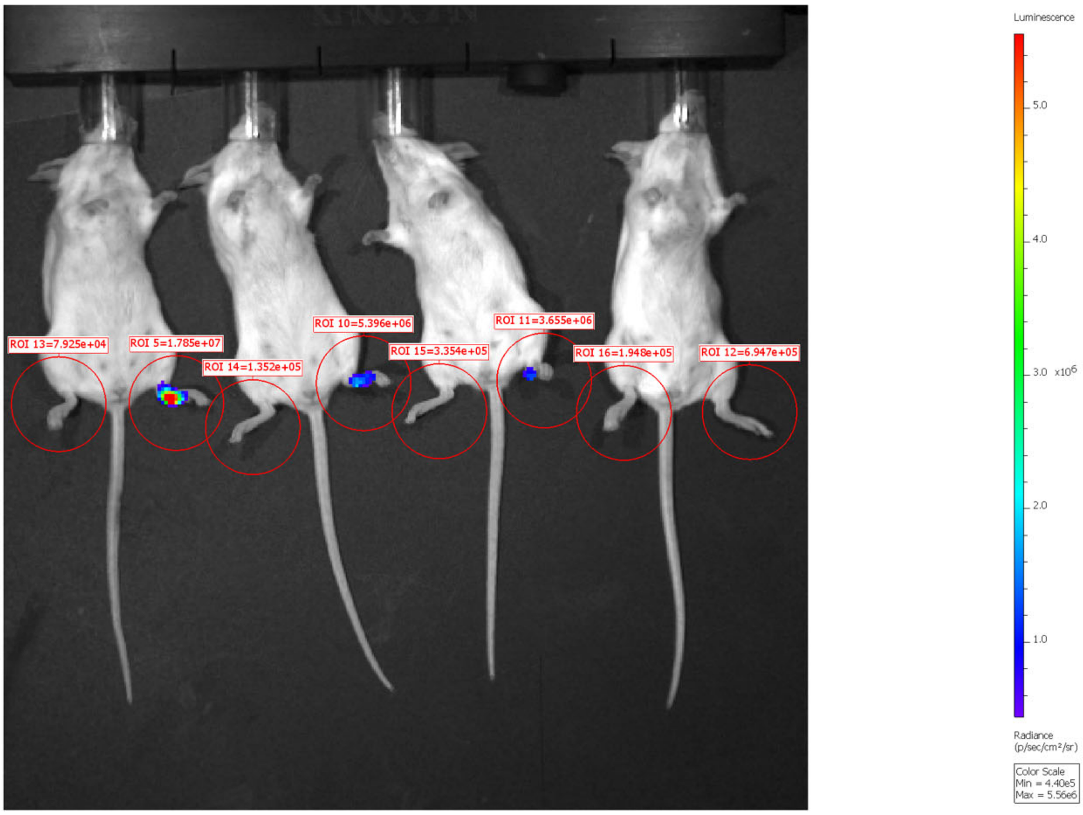

Supplement: Supplementary file 1 [file microorganisms-14-01135-s001.zip › microorganisms-4204300-supplementary.pdf]
